# The distribution of autistic traits across the autism spectrum: evidence for discontinuous dimensional subpopulations underlying the autism continuum

**DOI:** 10.1186/s13229-019-0275-3

**Published:** 2019-05-27

**Authors:** Ahmad Abu-Akel, Carrie Allison, Simon Baron-Cohen, Dietmar Heinke

**Affiliations:** 1 Institut de Psychologie, Université de Lausanne, Quartier UNIL-Mouline, Géopolis, CH-1015 Lausanne, Switzerland; 2 Autism Research Centre, Department of Psychiatry, University of Cambridge, Cambridge, UK; 3 School of Psychology, University of Birmingham, Birmingham, UK

## Abstract

**Background:**

A considerable amount of research has discussed whether autism and psychiatric/neurodevelopmental conditions in general are best described categorically or dimensionally. In recent years, finite mixture models have been increasingly applied to mixed populations of autistic and non-autistic individuals to answer this question. However, the use of such methods with mixed populations may not be appropriate for two reasons: First, subgroups within mixed populations are often skewed and thus violate mixture models assumptions, which are based on weighted sum of Gaussian distributions. Second, these analyses have, to our knowledge, been solely applied to enriched samples, where the prevalence of the clinical condition within the study sample far exceeds epidemiological estimates.

**Method:**

We employed a dual Weibull mixture model to examine the distribution of the Autism Spectrum Quotient scores of a mixed sample of autistic and non-autistic adults (*N* = 4717; autism = 811), as well as of a derived sample (from the enriched sample; *N* = 3973; autism = 67) that reflects the current prevalence of autism within the general population.

**Results:**

In a mixed autistic and non-autistic population, our model provided a better description of the underlying structure of autistic traits than traditional finite Gaussian mixture models and performed well when applied to a sample that reflected the prevalence of autism in the general population. The model yielded results, which are consistent with predictions of current theories advocating for the co-existence of a mixed categorical and dimensional architecture within the autism spectrum.

**Conclusion:**

The results provide insight into the continuum nature of the distribution of autistic traits, support the complementary role of both categorical and dimensional approaches to autism spectrum condition, and underscore the importance of analyzing samples that reflect the epidemiological prevalence of the condition. Owing to its flexibility to represent a wide variety of distributions, the Weibull distribution might be better suited for latent structure studies, within enriched and prevalence-true samples.

**Electronic supplementary material:**

The online version of this article (10.1186/s13229-019-0275-3) contains supplementary material, which is available to authorized users.

## Background

Autism spectrum condition (hereafter autism) is a neurodevelopmental condition that affects 1 in 59 children [1]. Autism is associated with difficulties in social communication and interaction, alongside restricted, repetitive pattern of behaviors and unusually narrow interests [2]. Current diagnostic practice conceptualizes autism categorically (i.e., absent or present). This conceptualization is supported by taxometric procedures identifying latent categorical structures within the population [3]. However, epidemiological evidence challenges such a taxonic point of view and suggests that autism phenotypes are not bound by conventional diagnostic thresholds, but rather blend imperceptibly with subclinical expressions within the general population, otherwise known as the broader autism phenotype [4–7]. Understanding the structure of the autism spectrum is important for improving diagnostic procedures, as well as for informing research design and the development of prognostic instruments [6–8]. To extend this line of research, we address shortcomings associated with the assumptions of analytical methods used to identify latent categorical structures within mixed populations and the epidemiological composition of the sample tested with these methods.

In recent years, univariate and multivariate finite normal mixture models, which are based on the weighted sum of Gaussian distributions [9, 10], have been applied to mixed populations data from children [11] and adults [7], to evaluate whether these models can detect discrete subgroups. Typically, such Gaussian mixture models [10] fit Gaussian distributions to a given dataset using an iterative search algorithm that varies the number of Gaussian distributions and their parameters. The resulting number of Gaussian distributions is usually interpreted as the number of subgroups or clusters in the data. The quality of fit for a given number of Gaussian distributions is evaluated with the likelihood criterion. Hence, if the fitting process was only guided by the likelihood criterion, the resulting number of subgroupings would be the same as the number of data points. Consequently, to estimate the most parsimonious number of subgroupings, the fitting process is controlled by a criterion balancing the number of subgroupings with the likelihood criterion.

We identify two major methodological limitations associated with the application of Gaussian mixture models in this important line of research. First, it has been noted that mixed populations, sampled from both clinical and community groups [12], including those of autistic and non-autistic populations [13], often consist of subgroups that are skewed, which might result from biases in ascertainment [3], or in psychometric properties of assessment scales [13]. Thus, Gaussian mixture models may not be appropriate for such data. Indeed, under such contexts, it has been recognized that a major drawback of Gaussian mixture models is the identification of spurious subgroups [9, 14, 15], probably precipitated by the tendency of these models to yield a better fit statistics as the number of Gaussian distributions (subgroups) increases (see also [16]). It has been suggested that information statistics (namely, the Akaike information criterion and the Bayesian information criterion) can be used to guide the identification of the “correct” number of subgroups generated by Gaussian mixture models applied to skewed data. However, it has been shown that these criteria, which also assume normal distributions, tend to either under- or overestimate the number of clusters due to their sensitivity to sample size and favoring highly parameterized models [17, 18]. Second, these analyses have, to our knowledge, been solely applied to enriched samples, i.e., where the prevalence of the clinical condition within the study sample far exceeds epidemiological estimates. This issue is of considerable importance as the results from study samples that do not reflect the epidemiological prevalence of the condition may not be clinically useful or credible [19], particularly when mixture modeling is used to establish a cutoff point that is subsequently used in clinical settings, or more broadly, when the results need to be generalizable.

The present study attempts to present solutions to these two methodological issues. Accordingly, the present study has two main aims. The first is to propose a model that can address the problem of spurious subgroupings generated by Gaussian mixture models when applied to samples consisting of skewed data. Specifically, we propose a dual distribution model, which combines two Weibull distributions [20] (see the “Methods” section). We chose the Weibull distribution because it has been shown to be advantageous when dealing with skewed distributions [21, 22], owing to its flexibility to represent a wide variety of distributions from nearly symmetric to highly skewed distributions [20]. The second aim is to see how the results of the dual Weibull mixture model compare between an enriched versus a prevalence-true sample (i.e., a sample that reflects the epidemiological prevalence of autism in the general population), which we generate from an enriched sample.

The proposed model is evaluated by examining the distribution of the Autism Spectrum Quotient scores (AQ) [4] of a large enriched mixed sample of autistic and non-autistic adults (*N* = 4717; autism = 811). The use of the AQ scores is predicated on the assumption that autistic traits lie on a continuously distributed spectrum, wherein variations within both the general population and clinically affected individuals are associated with common underlying genetic influences [23, 24]. Given the potential for sex-specific differences in the manifestation of autistic phenotypes [25, 26], the model is also applied to the distributions of autistic traits in the male and female subsamples. Finally, the model is evaluated within a subsample that reflects the current prevalence of autism within the general population [1].

## Methods

### Participants

This is a convenience sample, collected online, and which has previously been described and analyzed [26] to address whether normative sex differences in the general population are also observed in autistic people in terms of autistic, systemizing and empathizing traits. Briefly, the sample consisted of 4717 autistic and neurotypical adults. The overall sample (*M*_age_ (SD) = 34.47(13.16), age range = 18–75) consisted of 3016 females and 1701 males. The neurotypical group (*N* = 3906; *M*_age_ (SD) = 34.43(13.15)) consisted of 2562 females and 1344 males. The autistic group (*N* = 811; *M*_age_ (SD) = 34.66(13.21)) consisted of 454 females and 357 male. The autistic individuals self-reported having a formal clinical diagnosis of an autism spectrum condition as follows: Asperger syndrome (*n* = 506), high-functioning autism (*n* = 41), autism (*n* = 11), pervasive developmental disorder (*n* = 15), and autism spectrum condition (participants who did not specify a subtype) (*n* = 238). As has been previously reported [26], participants were excluded from both groups if they reported any of the following diagnoses/conditions: bipolar disorder, epilepsy, schizophrenia, attention-deficit/hyperactivity disorder, obsessive-compulsive disorder, learning disability, an intersex/transsexual condition, or psychosis.

There were no significant age differences between the autistic and neurotypical groups (*F* (1, 4715) = 0.20, *p* = 0.65), or between the males and females of the autistic (*F* (1, 809) = 0.17, *p* = 0.68) or the neurotypical (*F* (1, 3904) = 0.00, *p* = 0.96) groups. However, gender distribution across the autistic and the neurotypical groups was significantly different (*χ*^2^ = 26. 91, df = 1, *p* < 0.001), such that females were overrepresented in both the neurotypical (*χ*^2^ = 379. 81, df = 1, p < 0.001) and autistic (*χ*^2^ = 11. 60, df = 1, *p* = 0.001) groups.

### Measures

#### The Autism Spectrum Quotient

This self-report questionnaire consists of 50 items that measure the presence of traits associated with the autism spectrum in individuals with average or above average IQ [4]. These traits comprise five domains and include communication, social skills, attention to detail, imagination, and attention switching. Each item is given a score of 0 or 1. Higher total scores indicate the presence of greater autistic tendencies. The AQ has good sensitivity in capturing variation in quantitative autistic traits along the autism spectrum [4, 6].

### Model

**Fig. 1:**
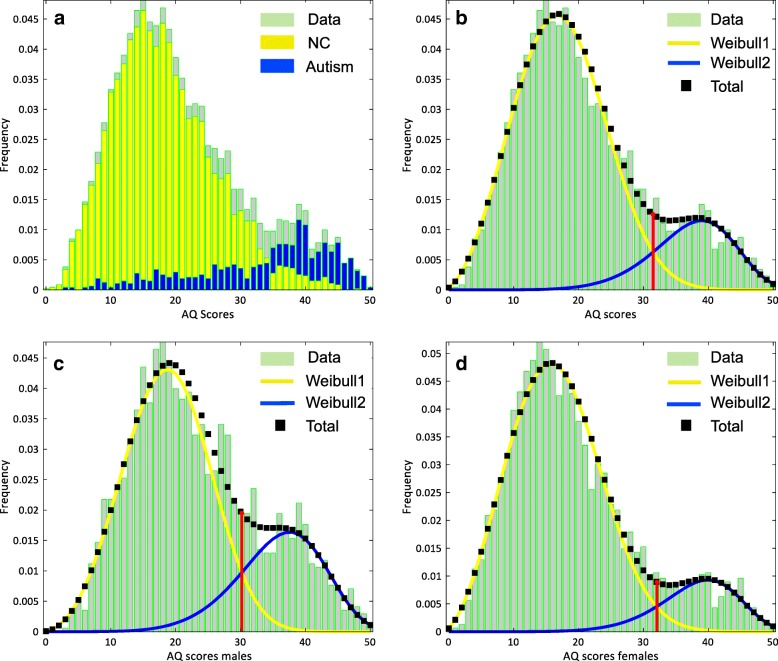
**a** The distribution of the AQ scores broken down according to diagnosis, neurotypical controls (NC; *N* = 3906), and autism groups (*N* = 811). **b** The histogram of the AQ scores of the overall sample (*N* = 4717) and the results of dual Weibull distribution model. **c** The histogram of the males’ AQ scores (NC = 1344; autism = 357) and the results of the dual Weibull distribution model. **d** The histogram of the females’ AQ scores (*N* = 2562; autism = 454) and the results of the dual Weibull distribution model. **b**–**d** The black dotted line represents the total model; the yellow and blue lines represent the Weibull1 (left) and Weibull2 (right) distributions, respectively. The red line indicates the intersection (cutoff) point between the two distributions. Each of the depicted plots (**b**–**d**) is of the bootstrapped sample whose threshold is closest to the mean threshold of all bootstrapped samples. We interpret the intersection point as the threshold score between the autistic individuals and the neurotypical controls

Here, we describe the development of a dual distribution model to assess the latent structure of a skewed distribution of AQ scores in a mixed adult population, including autistic and non-autistic individuals. We propose that the Weibull distribution could be the best description of the AQ score distribution, since a visual inspection of the histogram of the AQ scores of the entire sample suggests that the distribution is positively skewed (see Fig. 1a in the "Results" section). However, from Fig. 1a, we can also see that the distribution of high AQ scores showed a small hump, suggesting that the skewed distribution is overlaid with another, yet negatively skewed Weibull distribution. Given that our sample consists of both autistic and neurotypical individuals, the positive and negative distributions can conceivably be linked to the neurotypical and autistic groups, respectively. This suggests that the overall distribution might consist of two non-normal distributions (see [27] for a similar approach, but in the context of assessing anti-thyroglobulin antibody positivity as a marker of chronic thyroiditis—also known as Hashimoto’s disease). Our observation for this bimodality in the overall distribution of the data is corroborated by the Hartigan’s dip statistic [28], which indicated that the distribution deviated significantly from a unimodal distribution (Hartigan’s dip = 0.023, *p* < 0.001—implemented in R Version 3.3.3), thus indicating the existence of multiple distinct subgroups (Additional file 1: Figure S1). Taken together, we accordingly describe the data with a two-component mixture model of Weibull distributions.

The first Weibull distribution can be written as follows:

1 $$ {f}_1(x)=\frac{\beta 1}{\eta 1}\times {\left(\frac{x}{\eta 1}\right)}^{\beta 1-1}\times {e}^{-{\left(\frac{x}{\eta 1}\ \right)}^{\beta 1}} $$

And the second distribution can be written as follows:

2 $$ {f}_2(x)=\frac{\beta 2}{\eta 2}\times {\left(\frac{x}{\eta 2}\right)}^{\beta 2-1}\times {e}^{-{\left(\frac{x}{\eta 2}\ \right)}^{\beta 2}} $$

For both distributions, the scale parameters (*η*_1,_ *η*_2_) are analogous to the standard deviation in a normal distribution. The shape parameters (*β*_1_, *β*_2_) reflect the skewness of the distributions, where for values smaller than 3, the distribution is skewed to left (negatively skewed), and for values larger than 3, the distribution is skewed to the right (positively skewed).

To reflect these observations regarding the overall shape of the AQ scores, we introduce a finite Weibull mixture model that combines both Weibull distributions through a weighted sum (see also [29]):

3 $$ m(x)=\left(1-w\right)\times {f}_1(x)+w\times {f}_2(x) $$

The final dual distribution model, where *m* represents the probability density function (PDF) of the mixture model, thus estimates the value of five parameters as follows: scale *η*_1_, shape parameter *β*_1_, scale *η*_2_, shape parameter *β*_2_, and weight (*w*).

Note that the weight parameter (*w*) (known also as the mixing probability parameter [29]), which weighs the contribution of the two distributions to the overall shape, was introduced to the model since it is not clear how much each of the distributions contributes to the overall distribution. So for each AQ score, the two distributions indicate the probability of belonging to one of the two groups, such that a low AQ score is more likely to be classified as of a neurotypical individual (1 − *w*), while a very high AQ score is more likely to be classified as of an autistic individual (*w*).

The intersection point of the densities of the two distributions, i.e., the cutoff point (*θ*), would indicate the point at which the probability of belonging to one of the groups changes (see [30] for computational details). This approach is consistent with the use of the cutoff points provided by finite mixture models to identify discrete classes or components. This has been applied, for example, in the demarcation of short and long white matter fiber tract classes [31] and the positivity of anti-thyroglobulin antibody—a marker of Hashimoto’s disease [27].

### Model fitting

We used the principle of maximum likelihood to measure the goodness of fit of the dual-distribution model:

4 $$ L\left(\underset{\_}{x},{\eta}_1,{\beta}_1,w,{\eta}_2,{\beta}_2\right)=\prod \limits_{i=1}^Nm\left({x}_i\right) $$

*x* is the vector of AQ scores (data). *N* is the number of data points. As the multiplication of small values results in numerical issues, we followed standard practice and used log-likelihoods.

### Statistical analysis

To find the best fitting parameters, we employed Matlab’s fminsearch [32]. In general, fminsearch is a special case of an iterative algorithm. The aim of this particular iterative algorithm is to determine a parameter setting for a given mathematical function (the likelihood criterion of our model), which produces a minimal/maximal function value. The algorithm determines this parameter setting by executing a number of calculation steps, which build on each other (i.e., an iterative algorithm). Due to the complexity of the likelihood criterion, this method is needed because the maximum likelihood value cannot be determined through a simple one-step calculation (as in linear regression, where the parameters (slope and intercept) can be found in one simple calculation without iterations). It is important to note that these iterative algorithms require a starting point (initial parameter values). In our model fitting, fminsearch was applied to find the maximum likelihood value for the model’s parameter values (scale parameters *η*_1_ and *η*_2_, shape parameters *β*_1_ and *β*_2_, and weight *w*). We determined the starting point by finding a parameter setting for the model, which roughly followed the histogram of the AQ scores using trial and error. Hence, fminsearch can be seen as refining search of our initial guess.

To estimate the 95% confidence interval of the parameters, we employed the bootstrap method [33], since it is an appropriate method to apply to non-normally distributed data as well as for the estimation of parameters that cannot be analytically computed directly from the data. We resampled the data 1000 times with *replacement* and fitted the model to each sample. Note, each sampled dataset has the same size as the original dataset, but due to replacement, it is possible that data points are repeated within the sample. For each parameter, we calculated the mean of the 1000 resamples and determined the 95% confidence interval from the resulting distribution of the 1000 resamples. This procedure was applied to the overall sample, as well as to the male-only and female-only subsamples.

The significance of the difference between the cutoff points (*θ*) generated for the male-only and female-only samples was evaluated with a *t* test based on 1000 bootstrap samples from each sex. Since the significance of the *t* test depends on the number of participants (i.e., bootstrap samples)—a value that can be chosen arbitrarily—we estimated the effect size in terms of Cohen’s *d*, which is independent of the number of participants. To illustrate the implications of this predicted effect size for empirical studies, we determined the number of participants required to find a significant difference with a good probability. In other words, we conducted a power analysis using the bootstrapped thresholds [34]. Additional file 1: Figure S2 shows how the number of participants is related to different levels of power. For a statistical power of 0.9, the results indicate the need for around 15 participants per group. We note that the small *N* is due to the very small variance of the cutoff points (*θ*).

Next, we compared our dual Weibull distribution model to alternative single and multiple mixture distribution models, with the log-likelihood chi-square difference test, which accounts for the difference in the model’s fits (i.e., the log-likelihood values) and the model’s complexity (i.e., number of parameters). Specifically, we compared our dual Weibull distribution model to single Gaussian and single Weibull distribution models, to the results of the unsupervised finite Gaussian mixture model-based approach of Figueiredo and Jain [10], and to the following dual mixture models: Gauss-Gauss, Gauss-Weibull, and Weibull-Gauss model.

Finally, since our sample does not reflect the epidemiological prevalence of autism in the general population, we applied our dual Weibull distribution model to a resampled population that reflected the 1 in 59 (1.69%) prevalence of autism within the general population [1]. To do so, the data were separated into the neurotypical and the autistic groups. Each group was then bootstrapped separately whereby the autistic group was subsampled to reflect the prevalence of 1 in 59 of the combined bootstrapped samples. Note that because the bootstrap uses sampling with *replacement,* the same neurotypical/autistic individual can appear more than once in their respective resampled datasets. The dual distribution model was then fitted to each bootstrapped dataset, and the resulting parameter values were averaged. Importantly, it turned out that the initial parameters for the fitting process of these data did not lead to sensible solutions. Therefore, we used the parameter values from a previous analysis of the overall data, apart from the value of the weight (*w*) parameter, to initialize fminsearch. Since the previous analyses showed that the weight reflected approximately the prevalence of autism in the sample, we chose the prevalence of 1 in 59 as the initial weight value for the model fitting of the prevalence-true sample (see [27, 35] for a similar approach in which disease prevalence was used to assign the mixing probability value).

## Results

The results are presented in two parts. First, we report the results for the overall, autism-enriched sample, in which the autistic individuals reflected 17.19% of the overall sample, or about 10 times the estimated prevalence within the general population [1]. We then report the results for the prevalence-true sample of 1 in 59.

### Results of the autism-enriched sample

Table 1 shows the mean AQ scores for the overall, autism-enriched sample, as well as the male- and female-only subsamples in the autistic and neurotypical groups.

**Table 1:** Mean Autism Spectrum Quotient (AQ) scores and standard deviations (SDs) by group and sex within the enriched sample

Enriched sample	Number	Mean AQ	SD
Autistic group	811	33.73	10.57
Autistic males	357	34.81	9.10
Autistic females	454	32.88	11.54
Neurotypical group	3906	18.16	7.84
Neurotypical males	1344	20.27	7.85
Neurotypical females	2562	17.06	7.61
Overall	4717	20.84	10.23
Overall males	1701	23.32	10.06
Overall females	3016	19.44	10.06

#### The dual Weibull distribution model

We applied the dual Weibull distribution model to the overall sample (model 1), to the male-only subsample (model 2), and to the female-only subsample (model 3). The results of the models 1–3 are presented in Table 2 and depicted in Fig. 1. The model revealed that the two distributions in all samples intersected, on average, between 30 and 32 on the AQ scale. We interpret the threshold (*θ*) as the cutoff score distinguishing between autistic and neurotypical individuals. In addition, the mean threshold separating the neurotypical group from the autistic group in the females-only sample (*θ*_females_ = 31.96) was higher than the threshold in the males-only sample (*θ*_males_ = 30.16). A *t* test based on 1000 bootstrap samples from each sex revealed that the thresholds were significantly different from each other (*t*_(df = 1998)_ = 27.64, *p* < 0.001; Cohen’s *d* = 1.24). Note that Cohen’s *d* reflects the effect size of the difference in the test statistics *t*.

**Table 2:** Parameters of the dual Weibull distribution models of the overall, male-only, and female-only samples

Parameter	Result*	Bootstrap: 95% confidence interval		
		Mean*	Lower bound	Upper bound
Model 1: Overall sample (*N* = 4717) -Log-likelihood = 17,219.28				
Scale (*η*_1_)	40.90	40.89	39.29	42.17
Shape (*β*_1_)	7.08	7.14	5.96	8.36
Weight (*w*)	0.82	0.82	0.78	0.85
Threshold (*θ*)	31.50	31.53	29.60	33.20
Scale (*η*_2_)	20.50	20.49	19.81	21.07
Shape (*β*_2_)	2.89	2.89	2.79	3.01
Model 2: Male sample (*N* = 1701) -Log-likelihood = 6228.31				
Scale (*η*_1_)	39.59	39.52	36.62	41.66
Shape (*β*_1_)	6.41	6.48	5.01	7.99
Weight (*w*)	0.73	0.72	0.61	0.80
Threshold (*θ*)	30.20	30.16	26.35	33.10
Scale (*η*_2_)	21.91	21.86	20.15	23.05
Shape (*β*_2_)	3.35	3.36	3.16	3.62
Model 3: Female sample (*N* = 3016) -Log-likelihood = 10,887.69				
Scale (*η*_1_)	41.75	41.62	39.31	43.35
Shape (*β*_1_)	7.62	7.66	5.69	9.72
Weight (*w*)	0.86	0.86	0.82	0.89
Threshold (*θ*)	32.10	31.96	29.30	34.20
Scale (*η*_2_)	19.63	19.60	18.83	20.26
Shape (*β*_2_)	2.77	2.78	2.66	2.92

*The result column shows the parameter values from the sample with the highest likelihood in the original data. The mean column shows the average parameter value from the 1000 resamples

#### Model comparisons

**Fig. 2:**
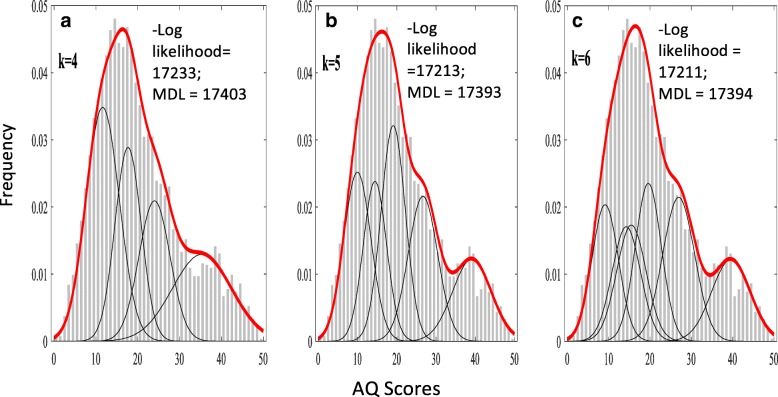
Subgrouping using the method by Figueiredo and Jain of finite mixture models with a weighted sum of Gaussian distributions. Depending on initialization, the models produced 4, 5, and 6 classes (*k* = 4, *k* = 5, *k* = 6) (number of Gaussian distributions; see black lines in panels **a**-**c**), which is likely due to compensation for deviations in the distribution of the data from the standard Gaussian distribution assumed by the model. Note that the model progressively improves the fit, as indicated by the decreasing likelihood values, with increasing number of components. However, the minimum description length (MDL), utilized by the Figueiredo and Jain method, indicated that the 5-component model is the most optimal model for the data

We indicated in the introduction that the application of standard finite mixture models with a weighted sum of Gaussian distributions could produce spurious subgrouping. Indeed, application of the popular method by Figueiredo and Jain [10] to our data detected four to six components (or subgroups) (see Fig. 2). This variation reflects the instability of the model and the (well-known) fact that the outcome of this method can depend on the order in which the data are presented. This problem results from the fact that Figueiredo and Jain method changes the model’s parameters each time a data point is presented to the model and that this change is based on the current parameter values. We note that none of the orders we tested produced fewer than four components. This method revealed that the 5-component model was the most optimal for the data, based on the minimum description length (MDL = 17,383), a formalization of Occam’s razor principle, which balances the model’s complexity with the model’s quality of fit (see Fig. 2). The log-likelihood chi-square difference test, comparing the likelihood values of the 5-component finite normal mixture model and our dual Weibull mixture model, was non-significant (*χ*^2^ = 12.56, df = 7, *p* = .084). This result suggests that the dual Weibull model is preferred, at least based on the principle of parsimony.

**Fig. 3:**
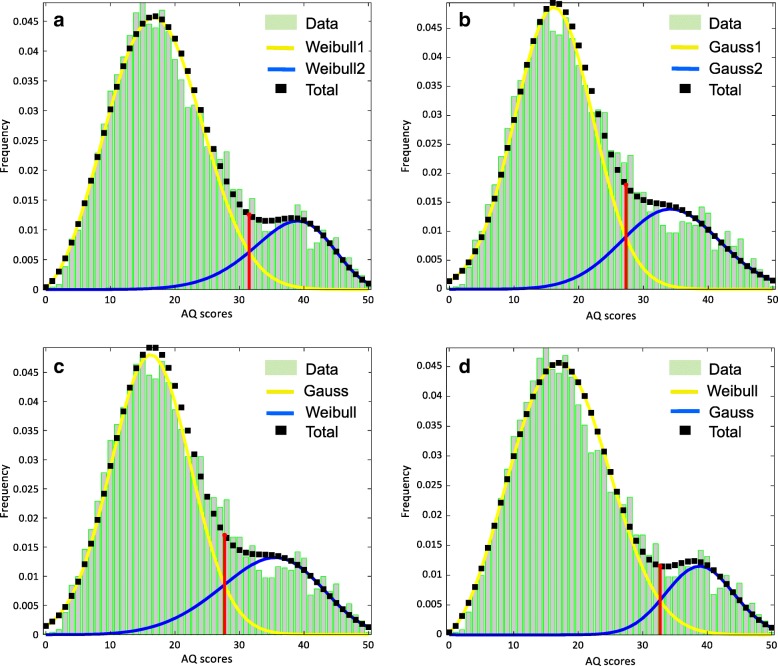
Comparison of the dual Weibull with the dual Gauss, Gaus-Weibull, and Weibull-Gauss distribution models. **a** The results of the dual Weibull distribution model (same as Fig. 1b). **b** The results of the dual Gauss distribution model. **c** The results of the Gauss-Weibull distribution model. **d** The results of the Weibull-Gauss distribution model. Each of the depicted plots (**a**–**d**) is of the bootstrapped sample whose threshold is closest to the mean threshold of all bootstrapped samples

In addition, we fitted three additional dual mixture models, using the same method as we fitted our Weibull-Weibull mixture model. These were a Gauss-Gauss model, a Gauss-Weibull model, and a Weibull-Gauss model (see Fig. 3; Table 3). The log-likelihood values, where smaller values indicate a better fit, suggested that our Weibull-Weibull model outperformed all models (all *p*s < .001) in the following order: Weibull-Weibull (−log-likelihood = 17,219.28) < Weibull-Gauss (−log-likelihood = 17,229.37) < Gauss-Weibull (−log-likelihood = 17,261.31) < Gauss-Gauss (−log-likelihood = 17,272.30). In addition, all the dual mixture models outperformed (all *p*s < .001) the single Gauss (−log-likelihood = 22,901.54) and the single Weibull (−log-likelihood = 17,393.10) distribution models (figures not shown).

**Table 3:** Parameters of the dual Gauss, Gauss-Weibull, and Weibull-Gauss distribution models for the overall sample

Parameter	Result*	Mean*	Bootstrap: 95% confidence interval	
			Lower bound	Upper bound
Model 4: Gauss + Gauss** (*N* = 4717) -Log-likelihood = 17,272.30				
Mean (*μ*_1_)	16.14	16.12	15.18	17.07
Std. dev. (*σ*_1_)	6.06	6.06	5.57	6.60
Weight (*w*)	0.74	0.74	0.65	0.81
Threshold (*θ*)	27.30	27.30	24.40	30.35
Mean (*μ*_2_)	34.19	34.17	30.87	37.20
Std. dev. (*σ*_2_)	7.50	7.48	6.11	8.74
Model 5: Gauss + Weibull (*N* = 4717) -Log-likelihood = 17,261.31				
Scale (*μ*)	16.24	16.18	15.10	16.91
Shape (*σ*)	6.15	6.13	5.56	6.57
Weight (*w*)	0.26	0.27	0.20	0.37
Threshold (*θ*)	27.70	27.56	24.10	30.05
Scale (*η*)	38.08	37.93	34.48	40.15
Shape (*β*)	5.18	5.19	3.88	6.41
Model 6: Weibull + Gauss -Log-likelihood = 17,229.37				
Scale (*η*)	20.92	20.88	20.05	21.57
Shape (*β*)	2.85	2.86	2.74	3.00
Weight (*w*)	0.85	0.85	0.80	0.88
Threshold (*θ*)	32.70	32.60	30.00	34.70
Mean (*μ*)	38.83	38.73	36.58	40.27
Std. dev. (*σ*)	5.25	5.28	4.44	6.32

*The result column shows the parameter values from the sample with the highest likelihood in the original data. The mean column shows the average parameter value from the 1000 resamples

**μ_1_ and *σ*_1,_ and *μ*_2_ and *σ*_2_ correspond to the parameters of Guass1 and Gauss2 distributions depicted in Fig. 3b

### Prevalence-true sample

In this section, we report the results of the dual Weibull model for the resampled population (*N* = 3973), reflecting the 1.69% prevalence of autism within the general population. Table 4 shows the mean AQ scores of the prevalence-true sample, as well as of its male- and female-only subsamples in the autistic and neurotypical groups.

**Table 4:** Mean Autism Spectrum Quotient (AQ) score and standard deviations (SDs) by group and sex within the prevalence-true sample

Prevalence-true Sample	Mean *N*	Mean AQ	SD
Autism group	67	33.76	1.30
Autistic males	29.36	35.15	1.30
Autistic females	37.64	35.16	1.01
Neurotypical group	3906	18.17	0.12
Neurotypical males	1344.44	18.17	0.17
Neurotypical females	2561.56	18.16	0.09
Overall	3973	18.43	0.12
Overall males	1373.80	20.59	0.21
Overall females	2599.20	17.29	0.15

**Fig. 4:**
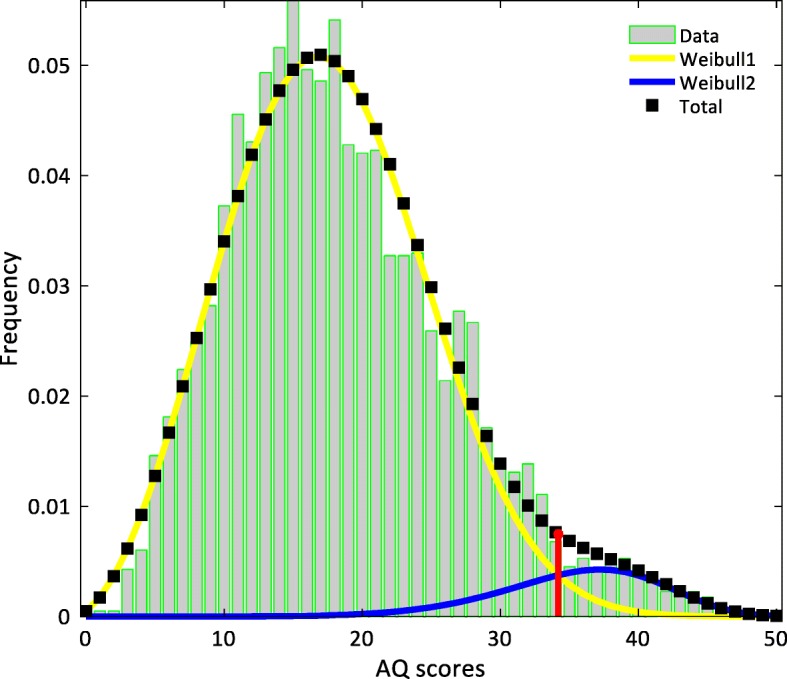
Histogram of the prevalence-true sample and the results of the dual Weibull distribution model. Black dotted line represents the total model; yellow and blue lines represent the Weibull1 and Weibull2 distributions, respectively. The red line indicates the intersection point between the two distributions. The depicted plot is of the bootstrapped sample whose threshold was closest to the mean threshold of all bootstrapped samples. We interpret the intersection point as the threshold score between autistic and neurotypical individuals, estimated at about 34 on the AQ scale

The results of the dual Weibull distribution model are presented in Table 5 and depicted in Fig. 4. As before, we interpret the intersection of the two distributions (*θ*_prevalence-true_ = 34.18) as the cutoff score distinguishing between autistic and neurotypical individuals. We note that this threshold is significantly higher than the mean threshold we observed for the enriched sample (*θ*_enriched_ = 31.53; *t*_(df = 1998)_ = 75.10; *p* < 0.001; Cohen’s *d* = 3.36). In addition, we note that the fit of this dual Weibull distribution was significantly a better fit than a model with a single Weibull distribution (−log-likelihood = 13,847.04; *χ*^2^ = 51.86, df = 3, *p* < .001).

**Table 5:** Parameters of the dual Weibull-Weibull distribution model of the prevalence-true sample

Parameter	Result*	Bootstrap: 95% confidence interval		
		Mean*	Lower bound	Upper bound
Model 7: Prevalence-true sample (*N* = 3973) -Log-likelihood = 13,821.11				
Scale (*η*_1_)	39.00	39.08	39.00	40.65
Shape (*β*_1_)	7.30	6.85	6.16	8.21
Weight (*w*)	0.94	0.94	0.93	0.96
Threshold (*θ*)	34.20	34.18	33.20	35.75
Scale (*η*_2_)	20.71	20.58	20.27	20.94
Shape (*β*_2_)	2.84	2.86	2.77	2.94

*The result column shows the parameter values from the sample with the highest likelihood. The mean column shows the average parameter value from the 1000 resamples

## Discussion

Finite normal mixture models have been increasingly applied to mixed populations of autistic and non-autistic individuals to ascertain the underlying structure of the autism spectrum. However, such mixed populations often consist of subpopulations with skewed distributions, which violate normal mixture models assumptions, which are based on the weighted sum of Gaussian distributions. Second, these analyses have, to our knowledge, been solely applied to enriched samples, where the prevalence of the clinical condition within the study sample far exceeds epidemiological estimates. We addressed these limitations in a mixed sample of autistic and non-autistic individuals. With respect to the first shortcoming, we proposed a dual Weibull distribution model, owing to its flexibility in accounting for a variety of distributions including both negatively and positively skewed distributions. We demonstrated that our dual Weibull distribution model outperformed alternative single (Gauss, Weibull) and dual (Table 3: Gauss-Gauss, Gauss-Weibull, and Weibull-Gauss) distribution models. In addition, it was more parsimonious and thus preferred over the 5-component structure recommended by the Figueiredo and Jain finite mixture model (see Fig. 2). With respect to the second shortcoming, we showed that our dual Weibull distribution model performed well when applied to the prevalence-true sample (which we generated from the enriched sample) and was superior to a model with a single Weibull distribution.

The results showed that the distribution of autistic traits reflects a dimensional structure, comprised of two components that reasonably reflected the nature of our mixed sample of autistic and non-autistic individuals, and thus may inform the debate pertaining to whether autism is best characterized as a category [3] or as a dimension [36]. We suggest that our results support the idea that both dimensional and categorical classification of autism need not be mutually exclusive [8, 37]. More specifically, the quantitative increases in AQ scores, within both and the autistic and non-autistic groups, may reflect a single dimension of, for example, genetic liability that underlies the autism spectrum condition [23, 24]. Yet, the two-component structure suggests that differences in the extent to which autistic traits are present can also be explained in terms of the absence/presence of the condition. This interpretation is consistent with recent conclusions advocating that both dimensional and categorical classifications of autism can be complementary [7, 8, 38, 39], as they may explain different aspects of the condition [38]. Taken together, these results reflect the spectrum nature of autistic traits within both the subclinical and clinical domains, and the substantial heterogeneity within the autistic spectrum [40]. Future research would be important to delineate further the contribution of dimensional and categorical classifications to the understanding of autism. However, we recommend that this need to be examined in representative populations that reflect the prevalence rate of the condition.

While the evaluation of the diagnostic properties of the AQ was not the point of this study, cutoff points seem to vary depending on the prevalence rate of autism within the population and sex. With respect to the prevalence rate, we observe that the cutoff point of the prevalence-true sample was significantly higher than the cutoff point of the enriched sample (*θ*_prevalence-true_ ~  34 vs. *θ*_enriched_ ~  32). While we emphasize that our findings should not be used to support one cutoff point over another, this difference suggests that *prevalence* can be a source of variation in the estimation of cutoff points. This is particularly important if the goal of the modeler is to establish a cutoff point that is subsequently used in clinical settings. Thus, minding the prevalence rate can thus boost the practical significance of findings in this line of research [19, 41].

Moreover, in considering the results of our model for both the male- and female-only samples, the cutoff point was significantly higher in the female- compared to the male-only sample. This is intriguing given that the mean AQ scores in both the autistic and non-autistic females are lower than the mean AQ scores of the autistic and non-autistic males. However, an inspection of the histogram (Additional file 1: Figure S3) suggests that non-autistic and autistic females occupy more the extreme ends of the AQ scale than their male counterparts. Thus, to the extent that these scores are expressions of the genetic liability to autistic traits [24], this difference in cutoff points is consistent with accounts suggesting that females require greater genetic liability, or etiological load, for the condition to be manifest [42, 43] and with reports showing that females need to show more severe problems to obtain a diagnosis [44–47]. It has recently been argued that sex differences in the etiology of autistic traits are minor and only detectable in large sample sizes [48]. However, the large effect size we observed for the difference in the cutoff points within the male and female samples suggests that this difference may prove important when comparing males and females in terms of ascertainment of diagnosis [49] and treatment response [47], for example.

Taken together, we infer from the observed threshold differences, between the enriched and prevalence-true samples, and between the male- and female-only samples, that subgroupings based on quantitative autistic traits of mixed autistic and non-autistic populations are susceptible to both sex and the prevalence of autism within the population. Therefore, our method to generate a sample reflecting the epidemiological prevalence of autism might be an important step forward in that it has the potential to increase the practical significance of this line of research which, to our knowledge, is solely conducted in enriched samples. This approach can be particularly useful knowing that sometimes it is necessary to have an enriched sample to perform various statistical analyses and that the relative rarity of clinical disorders within population samples would need to be very large to facilitate specific investigations within clinical populations. Nonetheless, future research with larger samples in which the overall prevalence also reflects the relative prevalence of autism in males and females is needed to fully assess the effect of prevalence on the results of finite mixture models in both normally and non-normally distributed data.

Methodologically, our statistical approach improves upon finite normal mixture models for the identification of subgroups of skewed distribution within a population. Specifically, we have demonstrated that our model has advantages compared to finite normal mixture models assuming weighted sum of Gaussian distributions, which are prone to yield spurious subgroupings when applied to such populations (see Fig. 2). This is consistent with previous simulation research showing that these models find it necessary to select more classes to better approximate the non-normal distribution of the latent structure to improve the fit [15, 16]. Therefore, we highlight that distribution misspecifications of the latent structure may lead to invalid results about the true structure of autistic traits within the population. As pointed out in the introduction, skewed distributions of the latent structures might result from biases in ascertainment [3] or in psychometric properties of assessment scales [13]. In considering our online sample, biases in ascertainment might explain the deviation from normality. The psychometric implications of ignoring latent distributional assumptions have recently been discussed [13] in the context of examining quantitative autistic traits of a mixed population measured with the Social Responsiveness Scale-Short Form [50]. Specifically, it has been argued that since this scale violates the assumption of an underlying latent normal distribution for the population, its psychometric properties, which are derived with techniques that assume normality, may therefore be invalid [13]. We propose that the application of Weibull distributions in the development of psychometric scales may help mitigate such shortfalls due to its flexibility in representing both symmetrical and asymmetrical distributions within the population [20]. However, since the results of finite mixture models depend on selecting the appropriate distribution for the latent structure [51], future studies should examine the applicability of other distributions that can handle skewed data, such as the lognormal [52] and skew-normal distributions [53].

It is noteworthy that the results of the dual Weibull mixture model raise the possibility that traditional analytic methods such as general linear models may not be adequate to perform statistical analyses on mixed, autistic, and non-autistic populations, as they are built on assumptions that do not reflect the asymmetric distribution of their autistic traits (see Martinez et al. [54] for an example of how to perform regression when the data consist of a mixture of components or distributions).

### Limitations

Our findings should be viewed with some limitations in mind. First, our results may be limited by the use of a single trait measure. Therefore, it is important for future research to replicate our findings with different instruments, such as the Social Responsiveness Scale [50, 55]. Second, our sample was collected online and therefore may have a sampling bias. Moreover, the diagnostic data are based on self report and were not clinically verified. However, clinical diagnoses of autism that are reported by online volunteers tend to be generally reliable [56]. Equally likely, there may be clinical but undiagnosed cases in the neurotypical sample. Third, while our analysis of the prevalence-true sample provides insight into the ecological validity of the distribution structure of autistic traits within the general population, larger samples are needed in order to also reflect the true male to female ratio of autism. However, we do not suspect that the overrepresentation of females in our sample to have affected our results, since the distributional structure was similar across the male- and female-only samples (see Fig. 1c, d). Finally, our dual Weibull distribution model was restricted to two distributions. This was based on our knowledge of the sample composition (autistic and non-autistic individuals) and the bimodality of the distribution. Of course, this does not preclude the presence of more components underlying the structure of autistic traits, and therefore, a more flexible model of Weibull distributions would be needed to determine if the sample comprises of more than two components. To the best of our knowledge, such a flexible model is not available for the *fminsearch* method we used to fit the models. However, we note that the fit of our dual Weibull distribution model was statistically indistinguishable from the 5-component model recommended by the finite normal mixture model (Fig. 2), and thus, it was preferred since it yielded a similar fit but with fewer components.

## Conclusion

Efforts aimed at integrating the categorical and dimensional perspectives of autism and other conditions are underway [38–40, 57]. However, with the increase in the popularity of finite mixture modeling to inform this debate, it is important for the modeler to ensure concordance between the model’s assumptions and the distribution of the latent structure within the population. Since a misspecification of the distribution of the latent structure could lead to spurious subgrouping (see Fig. 2), we caution that finding the best fitting mixture, particularly with the use of flexible finite mixture modeling [10], is not necessarily equivalent to finding the optimal partition for a given dataset [58]. Owing to its flexibility to represent a wide variety of distributions, the Weibull distribution might be better suited for latent structure studies, within enriched and prevalence-true populations. In addition, investigations concerned with the structure of the autism spectrum must also heed the influence of prevalence and sex on the model’s results to buttress its practical significance. With these considerations in mind, a multidimensional space that maps core features of autism would ultimately be needed to more precisely reflect the heterogenic nature of autism and the underlying structure of its spectrum.

## Additional file

Additional file 1: **Figure S1.** Hartigan’s dip test of unimodality. **Figure S2.** Power analysis of threshold comparison between the male and female samples. **Figure S3.** The distributions of Autism Spectrum Quotient (AQ) scores of the males and females with and without an autism spectrum condition within the overall enriched-sample (*N* = 4717). (DOCX 183 kb)

## Abbreviations

AQ: Autism Spectrum Quotient
NC: Neurotypical controls

## References

[1] Baio J, Wiggins L, Christensen DL, Maenner MJ, Daniels J, Warren Z (2018). Prevalence of autism spectrum disorder among children aged 8 years - autism and developmental disabilities monitoring network, 11 sites, United States, 2014. MMWR Surveill Summ.

[2] APA. Diagnostic and statistical manual of mental disorders (DSM-5®). Washington: American Psychiatric Association; 2013.

[3] Frazier TW, Youngstrom EA, Sinclair L, Kubu CS, Law P, Rezai A (2010). Autism spectrum disorders as a qualitatively distinct category from typical behavior in a large, clinically ascertained sample. Assessment..

[4] Baron-Cohen S, Wheelwright S, Skinner R, Martin J, Clubley E (2001). The autism-spectrum quotient (AQ): evidence from Asperger syndrome/high-functioning autism, males and females, scientists and mathematicians. J Autism Dev Disord.

[5] Austin EJ (2005). Personality correlates of the broader autism phenotype as assessed by the Autism Spectrum Quotient (AQ). Personal Individ Differ.

[6] Grove R, Baillie A, Allison C, Baron-Cohen S, Hoekstra RA (2014). The latent structure of cognitive and emotional empathy in individuals with autism, first-degree relatives and typical individuals. Molecular Autism..

[7] Grove R, Baillie A, Allison C, Baron-Cohen S, Hoekstra RA (2015). Exploring the quantitative nature of empathy, systemising and autistic traits using factor mixture modelling. Br J Psychiatry.

[8] Peralta V, Cuesta MJ (2007). A dimensional and categorical architecture for the classification of psychotic disorders. World Psychiatry.

[9] McLachlan GJ, Peel D (2000). Finite mixture models.

[10] Figueiredo MA, Jain AK (2002). Unsupervised learning of finite mixture models. IEEE Trans Pattern Anal Mach Intell.

[11] Eagle RF, Romanczyk RG, Lenzenweger MF (2010). Classification of children with autism spectrum disorders: a finite mixture modeling approach to heterogeneity. Res Autism Spectr Disord.

[12] Markon KE, Krueger RF (2006). Information-theoretic latent distribution modeling: distinguishing discrete and continuous latent variable models. Psychol Methods.

[13] Kaat AJ, Farmer C (2017). Commentary: lingering questions about the Social Responsiveness cale short form. A commentary on Sturm et al. (2017). J Child Psychol Psychiatry.

[14] Franczak BC, Browne RP, McNicholas PD (2014). Mixtures of shifted asymmetric Laplace distributions. IEEE Trans Pattern Anal Mach Intell.

[15] Guerra-Pena K, Steinley D (2016). Extracting spurious latent classes in growth mixture modeling with nonnormal errors. Educ Psychol Meas.

[16] Bauer DJ, Curran PJ (2003). Distributional assumptions of growth mixture models: implications for overextraction of latent trajectory classes. Psychol Methods.

[17] Fossati A, Citterio A, Grazioli F, Borroni S, Carretta I, Maffei C (2005). Taxonic structure of schizotypal personality disorder: a multiple-instrument, multi-sample study based on mixture models. Psychiatry Res.

[18] Zhang H, Huang Y (2015). Finite mixture models and their applications: a review. Austin Biometrics Biostatistics.

[19] Abu-Akel A, Bousman C, Skafidas E, Pantelis C (2018). Mind the prevalence rate: overestimating the clinical utility of psychiatric diagnostic classifiers. Psychol Med.

[20] Johnson NL, Kotz S, Balakrishnan N (1994). Continuous univariate distributions.

[21] Dwidayati N, Kartiko SH, Subanar (2013). Estimation of the parameters of a mixture Weibull model for analyze cure rate. Appl Math Sci.

[22] Narbutas V, Lin Y-S, Kristan M, Heinke D (2017). Serial versus parallel search: A model comparison approach based on reaction time distributions. Visual Cognition.

[23] Lundstrom S, Chang Z, Rastam M, Gillberg C, Larsson H, Anckarsater H (2012). Autism spectrum disorders and autistic like traits: similar etiology in the extreme end and the normal variation. Arch Gen Psychiatry.

[24] Robinson EB, Koenen KC, McCormick MC, Munir K, Hallett V, Happe F (2011). Evidence that autistic traits show the same etiology in the general population and at the quantitative extremes (5%, 2.5%, and 1%). Arch Gen Psychiatry.

[25] Kirkovski M, Enticott PG, Fitzgerald PB (2013). A review of the role of female gender in autism spectrum disorders. J Autism Dev Disord.

[26] Baron-Cohen S, Cassidy S, Auyeung B, Allison C, Achoukhi M, Robertson S (2014). Attenuation of typical sex differences in 800 adults with autism vs. 3,900 controls. PLoS One.

[27] Nakashima E, Fujii Y, Imaizumi M, Ashizawa K (2008). Finite mixture models in assessing anti-thyroglobulin antibody positivity as a marker of chronic thyroiditis. Jpn J Biometrics.

[28] Hartigan JA, Hartigan PM (1985). The dip test of unimodality. Ann Stat.

[29] Razali AM, Salih AA (2009). Combining two Weibull distributions using a mixing parameter. Eur J Sci Res.

[30] Trang NV, Choisy M, Nakagomi T, Chinh NT, Doan YH, Yamashiro T (2015). Determination of cut-off cycle threshold values in routine RT-PCR assays to assist differential diagnosis of norovirus in children hospitalized for acute gastroenteritis. Epidemiol Infect.

[31] Ecker C, Andrews D, Dell'Acqua F, Daly E, Murphy C, Catani M (2016). Relationship between cortical gyrification, white matter connectivity, and autism Spectrum disorder. Cereb Cortex.

[32] Nelder JA, Mead R (1965). A simplex method for function minimization. Comput J.

[33] Efron B, Tibshirani R (1993). An introduction to the bootstrap.

[34] Wilcox RR (2017). An introduction to robust estimation and hypothesis testing.

[35] Thompson TJ, Smith PJ, Boyle JP (1998). Finite mixture models with concomitant information: assessing diagnostic criteria for diabetes. Appl Stat.

[36] Wiggins LD, Robins DL, Adamson LB, Bakeman R, Henrich CC (2012). Support for a dimensional view of autism spectrum disorders in toddlers. J Autism Dev Disord.

[37] Ousley O, Cermak T (2014). Autism spectrum disorder: defining dimensions and subgroups. Curr Dev Disord Rep.

[38] Jalbrzikowski M, Ahmed KH, Patel A, Jonas R, Kushan L, Chow C (2017). Categorical versus dimensional approaches to autism-associated intermediate phenotypes in 22q11.2 microdeletion syndrome. Biol Psychiatry Cogn Neurosci Neuroimaging.

[39] Krueger RF, Kotov R, Watson D, Forbes MK, Eaton NR, Ruggero CJ (2018). Progress in achieving quantitative classification of psychopathology. World Psychiatry.

[40] Lai MC, Lombardo MV, Chakrabarti B, Baron-Cohen S (2013). Subgrouping the autism “spectrum”: reflections on DSM-5. PLoS Biol.

[41] Fusar-Poli P, Hijazi Z, Stahl D, Steyerberg EW (2018). The science of prognosis in psychiatry: a review. JAMA Psychiatry.

[42] Jacquemont S, Coe BP, Hersch M, Duyzend MH, Krumm N, Bergmann S (2014). A higher mutational burden in females supports a “female protective model” in neurodevelopmental disorders. Am J Hum Genet.

[43] Robinson EB, Lichtenstein P, Anckarsater H, Happe F, Ronald A (2013). Examining and interpreting the female protective effect against autistic behavior. Proc Natl Acad Sci U S A.

[44] Begeer S, Mandell D, Wijnker-Holmes B, Venderbosch S, Rem D, Stekelenburg F (2013). Sex differences in the timing of identification among children and adults with autism spectrum disorders. J Autism Dev Disord.

[45] Murray AL, Booth T, Auyeung B, McKenzie K, Kuenssberg R. Investigating sex bias in the AQ-10: a replication study. Assessment. 2017: 1073191117733548. 10.1177/1073191117733548 28954527

[46] Rutherford M, McKenzie K, Johnson T, Catchpole C, O'Hare A, McClure I (2016). Gender ratio in a clinical population sample, age of diagnosis and duration of assessment in children and adults with autism spectrum disorder. Autism.

[47] Singer L (2015). Thoughts about sex and gender differences from the next generation of autism scientists. Molecular Autism.

[48] de Zeeuw EL, van Beijsterveldt CEM, Hoekstra RA, Bartels M, Boomsma DI (2017). The etiology of autistic traits in preschoolers: a population-based twin study. J Child Psychol Psychiatry.

[49] Kopp S, Gillberg C (2011). The Autism Spectrum Screening Questionnaire (ASSQ)-Revised Extended Version (ASSQ-REV): an instrument for better capturing the autism phenotype in girls? A preliminary study involving 191 clinical cases and community controls. Res Dev Disabil.

[50] Sturm A, Kuhfeld M, Kasari C, McCracken JT (2017). Development and validation of an item response theory-based Social Responsiveness Scale short form. J Child Psychol Psychiatry.

[51] Elmahdy EE (2017). Modelling reliability data with finite Weibull or lognormal mixture distributions. Appl Math Inform Sci.

[52] Dumonceaux R, Antle CE (1973). Discrimination between the log-normal and the Weibull distributions. Technometrics..

[53] Azzalini A (1985). A class of distributions which includes the normal ones. Scand J Stat.

[54] Martinez GD, Bolfarine H, Salinas H (2017). Bimodal regression model. Revista Colombiana de Estadística.

[55] Constantino JN, Gruber CP (2005). Social Responsiveness Scale (SRS).

[56] Lee H, Marvin AR, Watson T, Piggot J, Law JK, Law PA (2010). Accuracy of phenotyping of autistic children based on internet implemented parent report. Am J Med Genet B Neuropsychiatr Genet.

[57] Lage D, Egli S, Riedel M, Strauss A, Moller HJ (2011). Combining the categorical and the dimensional perspective in a diagnostic map of psychotic disorders. Eur Arch Psychiatry Clin Neurosci.

[58] Melnykov V, Maitra R (2010). Finite mixture models and model-based clustering. Statistics Surveys.

