# Supplementary material for: The distribution of autistic traits across the autism spectrum: evidence for discontinuous dimensional subpopulations underlying the autism continuum
Source: Mol Autism. 2019 May 27;10:24. doi: 10.1186/s13229-019-0275-3 (PMC6537408; doi:10.1186/s13229-019-0275-3)
Supplement: Supplementary file 1 — Figure S1. Hartigan’s dip test of unimodality. Figure S2. Power analysis of threshold comparison between the male and female samples. Figure S3. The distributions of Autism Spectrum Quotient (AQ) scores of the males and females with and without an autism spectrum condition within the overall enriched-sample (N = 4717). (DOCX 183 kb) [file 13229_2019_275_MOESM1_ESM.docx]

**Supplementary Information**

**The distribution of autistic traits across the autism spectrum:**

**Evidence for discontinuous dimensional subpopulations**

**underlying the autism continuum**

Ahmad Abu-Akel^1^, Carrie Allison^2^, Simon Baron-Cohen^2^, Dietmar Heinke^3^

^1^ Institut de Psychologie, Université de Lausanne, Lausanne, 1015, Switzerland

^2^ Autism Research Centre, Department of Psychiatry, University of Cambridge, Cambridge, United Kingdom

^3^ School of Psychology, University of Birmingham, Birmingham, United Kingdom

Correspondence: Ahmad Abu-Akel. Université de Lausanne, Institut de Psychologie, Quartier UNIL-Mouline, Géopolis, CH-1015 Lausanne. Email: ahmad.abuakel@unil.ch

**Hartigan’s Dip Test of Unimodality**

We employed the Hartigan’s dip statistic to test for multimodality in the distribution of the AQ scores, where a significant deviation from unimodality is assumed to be indicative of multiple groups based on their scores. When examining the distribution of AQ scores for the entire sample (see Figure 1S), we found that the distribution deviated significantly from a unimodal distribution (Hartigan’s Dip = 0.023, P < 0.001), thus indicating the existence of multiple subgroups.


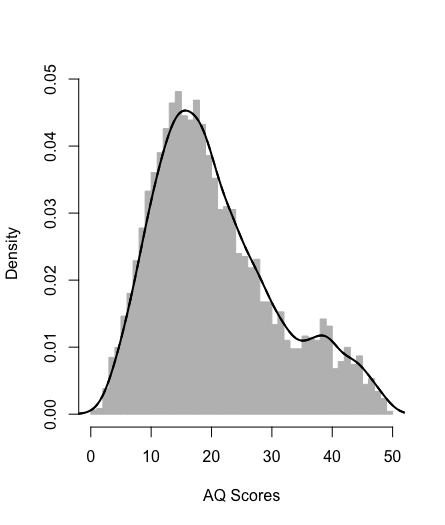

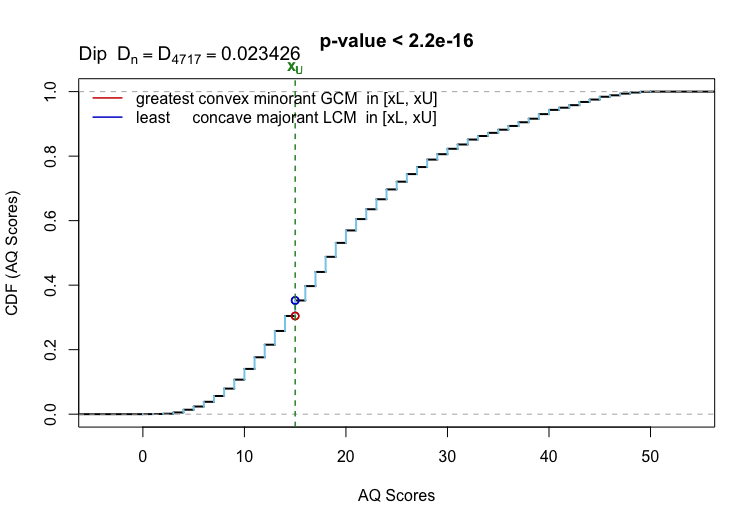


**Figure 1S**. Hartigan’s dip test of unimodality. **Left panel** visualizes the histogram and the density plot of the data (N = 4717). The **right panel** displays the results of the *dip statistic*, which is defined as the maximum difference between an empirical distribution function (CDF) and the unimodal distribution function that minimizes that maximum difference.

**Power analysis of threshold comparison between the male and female samples**

**Figure 2S**. Power analysis of threshold comparison between the male and female samples. For this graph, the bootstrapped thresholds from the two sexes were used to assess the power of a t-test between the two thresholds. To determine the power for a given number of participants, a subset of thresholds were taken (simulating the number of participants) and a t-test was performed. This process was repeated 1,000 times and the frequency of a significant outcome (the probability of finding a significant effect, i.e., power) was calculated. These steps were performed for 2, 6, 8, … 42 participants. As indicated by the red dashed line, a power of 0.90 was achieved with around 15 participants. This small number of participants is sufficient owed to the very small variances in the thresholds of two groups.

**The distributions of Autism Spectrum Quotient (AQ) scores stratified**

**in terms of diagnosis and sex**


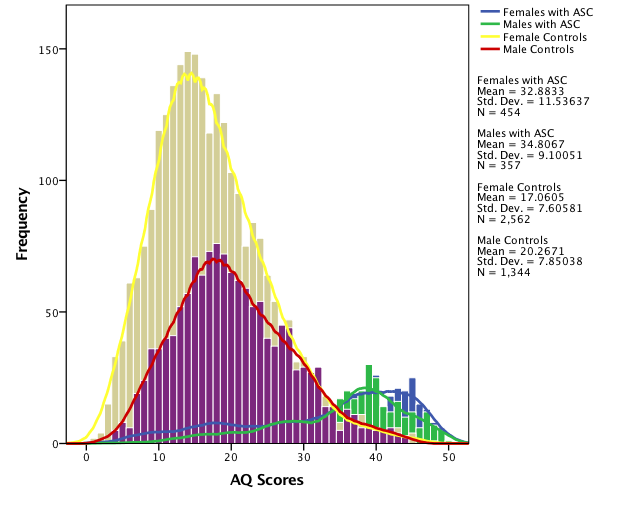


**Figure 3S**. The distributions of Autism Spectrum Quotient (AQ) scores of the males and females with and without an autism spectrum condition within the overall enriched-sample (N = 4717). Note that although the mean AQ score of the autistic females (blue bars) is smaller than the autistic males, autistic females are more frequently represented at the extreme of the AQ scale (AQ > 40).

.
